# Multicentric Spinal Tuberculosis with Sternoclavicular Joint Involvement: A Rare Presentation

**DOI:** 10.1155/2014/685406

**Published:** 2014-10-20

**Authors:** Balaji Saibaba, Umesh Kumar Meena, Prateek Behera, Ramesh Chand Meena

**Affiliations:** ^1^Department of Orthopaedics, SMS Medical College, Jaipur, Rajasthan 302004, India; ^2^Department of Orthopaedics, SMS Medical College and Hospital, Jaipur 302004, India

## Abstract

*Background*. Tuberculosis is a chronic disease which may have varied presentations. Though pulmonary tuberculosis is the commonest, extrapulmonary tuberculosis involving skeletal system is often seen. Individuals with poor nourishment and immunological status are especially susceptible for disseminated and multicentric tuberculosis. *Case Report*. We here present a case of tuberculosis involving multiple anatomical locations in an immune-competent patient which was diagnosed with radiological studies and confirmed with histological examination. Patient was put on multidrug antitubercular therapy and responded well to the treatment with improvement in clinical and radiological picture. *Clinical Relevance*. This report of a rare case makes us aware of the varied presentations which tuberculosis can present with. It should be kept as a differential diagnosis in patients with cough and fever but not responding to conventional treatment. This is even more important in countries with poor socioeconomic conditions.

## 1. Introduction

Tuberculosis (TB) is still a very common disease in developing countries [1]. Pulmonary tuberculosis is the commonest form of tuberculosis but patients may present with lesions in location not involving the lungs. Skeletal tuberculosis constitutes around 10% of the extrapulmonary cases. Spinal tuberculosis is most common and a dangerous form of skeletal tuberculosis in adults. The lower thoracic and upper lumbar vertebrae are the most common sites of involvement. The cervical spine is rarely affected; cervical spine involvement occurs in approximately 0.03% of all tuberculosis cases [1, 2]. Isolated cervical spine [1] or sternoclavicular joint involvement [2, 3] also has been reported in literature but multicentric involvement is extremely rare [4–6]. Involvement of sternoclavicular joint along with spinal tuberculosis has never been reported previously in English literature. This case report describes a rare type of tuberculosis involving multiple anatomical structures (i.e., atlanto-axial junction, dorsal spine, and sternoclavicular joint with concomitant pulmonary tuberculosis) which we can hence label as multicentric tuberculosis. It was treated successfully with multidrug antitubercular therapy.

## 2. Case Report

**Figure 1:**
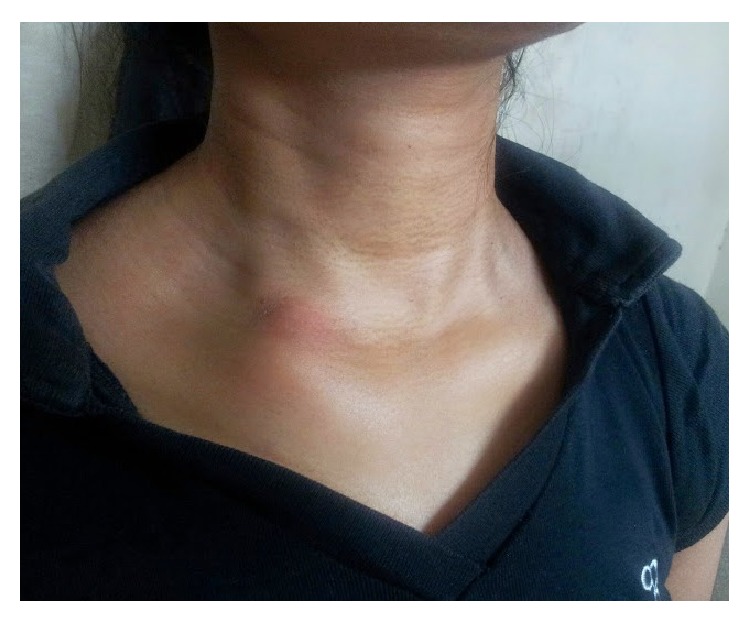
Clinical picture showing erythematous swelling over right sternoclavicular joint region.

**Figure 2:**
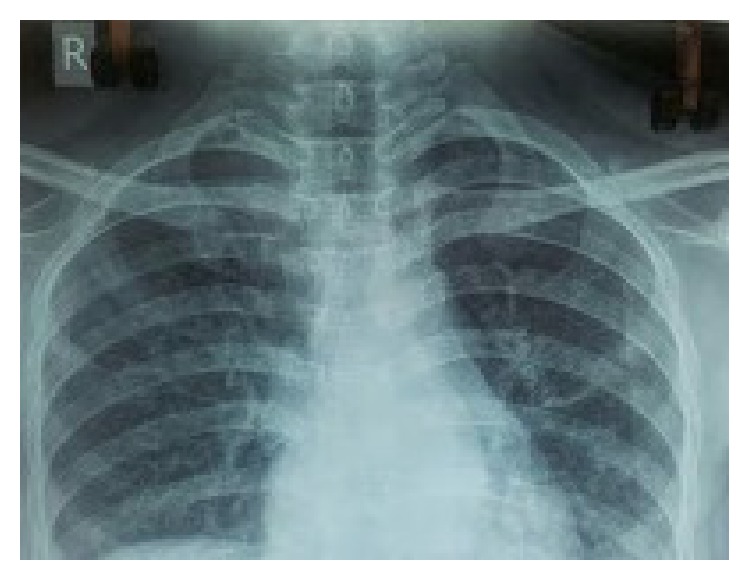
Radiology of patient showing diffuse pulmonary infiltrate along with destruction with sclerosis on the medial end of the right clavicle.

**Figure 3:**
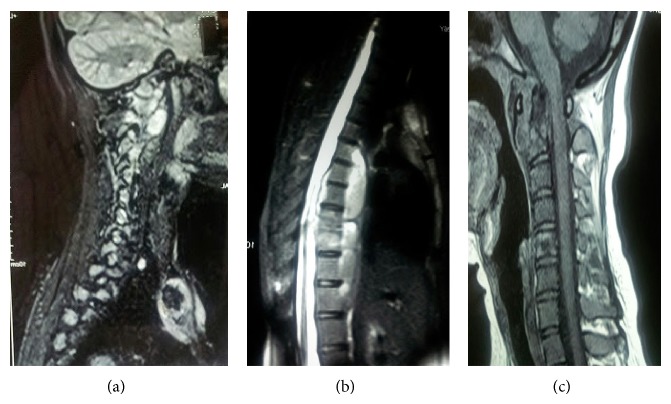
MRI images showing involvement of sternoclavicular joint along with dorsal and upper cervical involvement.

A 24-year-old unmarried female presented to the outpatient clinic with painful swelling of the right sternoclavicular joint of 2-month duration without any discharging sinus (Figure 1). The swelling was gradually increasing in size and was accompanied with mild pain. The pain was dull, continuous, and limited to the site of the lesion. She also complained of neck stiffness and pain on neck movements. There was no history of any injury. History of cough, weight loss, night cries (severe pain at night), and low grade fever was present for the past 4 months. There was no history of previous tuberculosis or contact with an open case of tuberculosis. She had been prescribed several antibiotics and analgesics at another centre but had no symptomatic improvement. On physical examination the swelling (2 × 3 cm) was present over right sternoclavicular joint and was associated with presence of mild tenderness, erythema, and local rise of temperature. Laboratory tests revealed haemoglobin of 10.4 gm%; total leukocyte count was 10.300/mm^3^. Her ESR was 34 mm in first hour. She was negative for HIV based on ELISA method. On radiographic evaluation there was destruction with sclerosis on the medial end of the right clavicle along with features of diffuse pulmonary infiltrate (Figure 2). MRI revealed bilateral upper lung lobe infiltrate with arthritis of right sternoclavicular joint, with regional fluid collection. A destruction of the atlanto-axial junction, D7-8 intervertebral disc space along with a pus collection from D5 to D8 region could also be appreciated (Figures 3(a), 3(b), and 3(c)). An early morning sputum sample was sent for Ziehl-Neelsen (ZN) staining and it came out positive suggesting the diagnosis of pulmonary tuberculosis. Fine needle aspiration of the right sternoclavicular lesion was done using a 22-gauge needle and sent for Gram staining, staining for acid-fast bacilli (AFB), histopathology, and cultures including a tubercular culture. The histologic picture was that of chronic inflammation with a caseating granuloma compatible with tuberculosis. The Ziehl-Neelsen stained smear also showed the presence of acid-fast bacilli (AFB), confirming the diagnosis of tuberculosis. The culture for* Mycobacterium tuberculosis* came out as negative. Antitubercular chemotherapy with four first line antitubercular drugs (rifampicin, isoniazid, ethambutol, and pyrazinamide) was started. The patient had a good clinical response within 6 weeks and was switched to three drugs (rifampicin, isoniazid, and ethambutol) after 3 months of therapy with four drugs. The clinical, haematological, and radiological parameters showed complete healing of the lesion after 1 year of treatment with ATT, which was further continued for a total duration of 18 months. After successfully completing the therapy for 18 months, the patient was followed up for 2 years and showed no recurrence of symptoms.

## 3. Discussion

Tuberculosis is a communicable disease caused by* M. tuberculosis*. It primarily affects the lungs and spreads by droplets and aerosols produced by coughing and sneezing by patients who are active cases of pulmonary tuberculosis. Extrapulmonary tuberculosis involving the skeletal system is not uncommon with skeletal tuberculosis accounting for 10% of extrapulmonary tuberculosis cases. Weight bearing joints involved in extrapulmonary tuberculosis are the spine, hip, and knees in the order of decreasing frequency. Involvement of the spine constitutes 60% of skeletal tuberculosis and the lower thoracic and upper lumbar vertebrae are more often affected; an involvement of the cervical spine is rare [1]. The usual pattern of spinal tuberculosis is of contiguous or continuous vertebral involvement but multicentric involvement is extremely rare [4–6]. Considering that the patients with skeletal tuberculosis may not present with the classical constitutional symptoms of tuberculosis, these patients may not be diagnosed early in the course of disease. Common predisposing factors for multicentric tuberculosis are immunocompromised status, intravenous drug use, diabetes mellitus, alcohol abuse, and hepatic cirrhosis [4, 7], but none of these risk factors was present in our case. The mode of involvement is most likely hematogenous, as suggested by its multicentric nature. Other routes of multicentric tuberculous involvement are direct inoculation, extension from adjacent bones or joints, and lymphogenous spread [4].

Cervical pain is common in young patients with or without history of trauma but a diagnosis of tuberculosis should be kept in mind in patients with atraumatic cervical pain of long duration and should be evaluated thoroughly if not relieved with initial therapy to prevent neurological complications. Our patient was also initially treated with this form of treatment without any improvement. Most patients with cervical lesions heal with adequate local support and antitubercular therapy, but surgical decompressio may required in presence of neurological impairment, instability, large cold abscess causing mechanical compressions or in refractory cases [1].

The occurrence of sternoclavicular tuberculosis is extremely rare and is difficult to diagnose on conventional radiographs. To prevent complications early diagnosis and treatment is essential. The differential diagnosis of sternoclavicular tuberculosis includes low grade pyogenic infection, rheumatoid disease, myeloma, or secondary metastatic deposits [1]. Poor response to ordinary antibiotic therapy should lead to suspicion of underlying tuberculosis especially in underdeveloped nations with poor living conditions and relevant investigations should be carried out. It has been suggested that all radiological and imaging modalities are complementary but MRI is probably the best imaging modality for early detection and diagnosis of sternoclavicular joint tuberculosis [8], and final diagnosis should be made only after confirmation with bacteriological or histological examination, as image findings are not fully reliable for differentiating spinal TB from other infections or neoplasms [9]. In our case, the MRI confirmed a lytic lesion on the medial end of the clavicle along with sclerosis and a collection of fluid around right sternoclavicular joint and could also suggest the possibility of concomitant pulmonary and spinal tuberculosis. Presence of pulmonary tuberculosis was confirmed by examination of the sputum of the patient. The final histological and microbiological confirmation of skeletal tuberculosis is by fine needle aspiration or open biopsy [3]. Diagnosis in our case too was confirmed by demonstration of AFB on ZN stain and by histological examination. In countries where tuberculosis is a common condition, a physician tends to attend to multiple cases of pulmonary tuberculosis. Some patients have skeletal tuberculosis with or without presence of concomitant pulmonary tuberculosis [6, 10–12]. It is very important that the treating physician picks up these cases early in the course of the disease. A physician needs to be aware that patients with pulmonary tuberculosis need to be investigated or screened for presence of skeletal involvement if they have any swelling in the neck or neck and back pain and should not be passed off as malaise.

Usual pulmonary TB treatment lasts from 9 to 12 months but in pulmonary TB if there is skeletal involvement prolonged antibiotic treatment is to be given as per majority consensus [13]. A 14–18-month duration of antitubercular therapy is required in spinal and sternoclavicular tuberculosis [3, 13]. Total duration of antitubercular therapy in our case was also of 18 months.

To conclude, a diagnosis of multicentric tuberculosis should be kept in mind in case of patients with atypical presentations in unusual locations with constitutional symptoms in endemic areas especially among undernourished and among those living in poor conditions. Examination of sputum should be performed in each case of suspected pulmonary tuberculosis. Imaging modalities should be supplemented with fine needle aspiration cytology or open biopsy to confirm the diagnosis. Timely diagnosis and treatment of multicentric tuberculosis will prevent further complications including paraplegia or deformity because of spinal tuberculosis or compression or erosion of the large blood vessels at the base of the neck and migration of the tuberculous abscess to the mediastinum in case of tuberculosis of sternoclavicular joint. Also the role of proper multidrug antitubercular therapy needs to be emphasized as tuberculosis can be very well managed with medications.
